# Increases in condomless chemsex associated with HIV acquisition in MSM but not heterosexuals attending a HIV testing center in Antwerp, Belgium

**DOI:** 10.1186/s12981-018-0201-3

**Published:** 2018-10-19

**Authors:** Chris Kenyon, Kristien Wouters, Tom Platteau, Jozefien Buyze, Eric Florence

**Affiliations:** 1 HIV/STI Unit, Institute of Tropical Medicine, Antwerp, Belgium; 2 Division of Infectious Diseases and HIV Medicine, University of Cape Town, Anzio Road, Observatory, Cape Town, 7700 South Africa; 3 Department of Clinical Sciences, Institute of Tropical Medicine, Antwerp, Belgium

**Keywords:** Chemsex, HIV, STIs, Syphilis, Chlamydia, Condomless sex

## Abstract

**Background:**

It has been speculated that the prevalence of chemsex is increasing in men who have sex with men and that this may be playing a role in the spread of HIV.

**Methods:**

We assessed if the prevalence of reported chemsex was increasing and if chemsex was associated with HIV infection in clients attending the ‘Helpcenter’, Antwerp, between 2011 and 2017. This is a HIV/STI testing center that offers HIV/STI testing to HIV-uninfected individuals from key populations including MSM.

**Results:**

We found an increase in the reporting of condomless sex associated with the use of a number of drugs, including ecstasy, amphetamines, GHB and cocaine in MSM (from 8 to 17%) but not in heterosexuals. Reporting condomless chemsex was associated with HIV infection (adjusted odds ratio 5.7 [95% confidence interval 3.2–10.4]).

**Conclusions:**

Our findings provide further evidence of the importance of asking MSM clients about the use of psychoactive substances during consultations and tailoring interventions such as pre exposure prophylaxis, more frequent STI screening and substance abuse counseling accordingly.

## Background

In the United Kingdom, the term “Chemsex” is used to describe sex under the influence of psychoactive drugs, mostly mephedrone, γ-hydroxybutyrate (GHB), γ-butyrolactone (GBL), and crystalized methamphetamine [1]. Whereas chemsex is observed in other countries, the products used to sustain, enhance or disinhibit sexual experiences may differ. No consensus European definition is available. Certain studies have found chemsex to be associated with a number of HIV risk factors such as multiple sexual partners, group sex and condomless sex in men who have sex with men (MSM) [2–4]. One study found an association between chemsex and incident sexually transmitted infections (STIs) in HIV-infected individuals [5]. Another study found an association between chemsex and incident hepatitis C infection in a pre-exposure prophylaxis cohort [6]. Other studies have however questioned how strong the association between chemsex and incident STIs is [1]. No studies that we are aware of have found chemsex to be a risk factor for HIV-infection. There is a widespread perception that the prevalence of this behavior is increasing in MSM but studies have shown that beliefs about the extent of chemsex use are exaggerated [7, 8]. The only published longitudinal data we could find was a small study that found an increase in reported chemsex use from 9/51 (18%) in 2013/14 to 41/101 (41%) in 2015 in Post Exposure Prophylaxis recipients in a single center in the United Kingdom [9]. ‘Antidote’, the United Kingdom’s only lesbian, gay, bisexual and transgender drug and alcohol support service has noted that the proportion of referrals due crystal meth, mephedrone and GHB/GBL increased from 3% in 2005 to 85% in 2012 [10]. We therefore undertook to investigate if [1] the prevalence of chemsex is increasing and [2] evaluate the relationship between chemsex and HIV in clients attending the ‘Helpcenter’, Antwerp, between 2011 and 2017. This is a center that offers free HIV/STI testing to HIV-uninfected individuals from key populations, including MSM, clients originating from high HIV prevalence regions (such as sub Saharan Africa), and those without access to healthcare (migrants and students). We used routinely collected data that asked clients if they had had condomless-sex under the influence of ecstasy/cocaine/amphetamines/GHB. Reporting yes to this question was defined as condomless-chemsex even though most studies define chemsex as sex under the influence of a somewhat different list of drugs-mephedrone, γ-hydroxybutyrate (GHB), γ-butyrolactone (GBL) or crystalized methamphetamine [2, 5, 6].

## Methods

We conducted an analysis of routinely collected data from clients attending our Helpcenter between 1 January 2011 and 6 February 2017. All clients attending the Helpcenter filled out a paper-based questionnaire prior to being evaluated clinically and tested. Whilst they were asked to complete all questions in the questionnaire, they were free to skip as many questions as they chose to. The questionnaire included the following two questions: (1) ‘Did you use any of the following drugs in the past 6 months?’ (2) ‘Have you had condomless sex under the influence of the following drugs?’ For both questions respondents could answer ‘yes’ or ‘no’ to four categories of drugs: ‘ecstasy/cocaine/amphetamines/GHB’, ‘alcohol’, ‘cannabis’ and ‘other’. We defined reporting condomless sex under the influence of ecstasy/cocaine/amphetamines/GHB as condomless-chemsex. Data were coded with a unique identifier and no personal identifiers were mentioned on the questionnaire. The unique identifier was used to link the clients questionnaires and laboratory results.

The questionnaire also asked clients to indicate if they had had sex with men, women or both. Men who reported having had sex with men or both men and women were classified as MSM whereas men and women who only reported having sex with the other gender were classified as heterosexuals. The number of women reporting sex with women (106) was small and these were therefore dropped from the analysis.

HIV testing was performed on venous blood using the Determine Combo HIV-1/2 Ag/Ab (Alere) test (4th generation). All reactive tests were confirmed using a standard confirmation algorithm with ELISAs and a confirmation test (INNO-LIA HIVI/II Score) [11].

### Statistical analysis

We investigated the association between HIV infection and chemsex at the individual level, but clients could make multiple visits to the Helpcenter. For this reason we needed to select which visit to use. For the HIV-seroconvertors we used the data measured at the visit when the individual first tested HIV-positive whereas for those who remained HIV-uninfected, we used the data from their first visit. Only a minority of clients tested HIV positive after the first visit (27/102 individuals testing HIV positive). The Chi square test for trend was used to assess for changes in proportions (by year, 2011 to 2016) reporting use of drugs and condomless sex under the influence of drugs. We used logistic regression to assess if there was an association between testing HIV-infected and reporting condomless chemsex whilst controlling for a range of confounders determined by a literature review. In the multivariate model we controlled for basic demographic confounders only (age, education and world region of origin). Simple logistic regression was also used to assess if there was an association between condomless chemsex and diagnoses of syphilis, chlamydia and gonorrhoea using the same methodology. All analyses were conducted separately for MSM and heterosexuals. All clients attending the Helpcenter signed an informed consent form for the collection and use of their behavioural and STI outcome data in analyses such as this one. This informed consent form was approved by the Ethics Committee of the University Hospital Antwerp.

## Results

The Helpcenter received a total of 11,220 visits by 6778 individuals between 1 January 2011 and 6 February 2017; 4270 (38.1%) of these were by 2121 MSM and 6950 (61.9%) by heterosexuals (2851 men and 1806 women). A total of 5000 (73.8%) clients made one visit, 992 (14.6%) two visits, 331 (4.9%) three visits and 455 (6.7%) more than three visits. The number of visits per year was stable at between 1604 and 2090 visits per year. The median age of clients was 34 (IQR 28-43). Over the course of the study, MSM were more likely than heterosexuals to report 5 or more partners in the previous 12 months (43.0% vs. 14.3%) and always using condoms in the previous 12 months (25.9% vs. 14.1%; P < 0.001 for both comparisons).

### Drug and condom usage

**Fig. 1:**
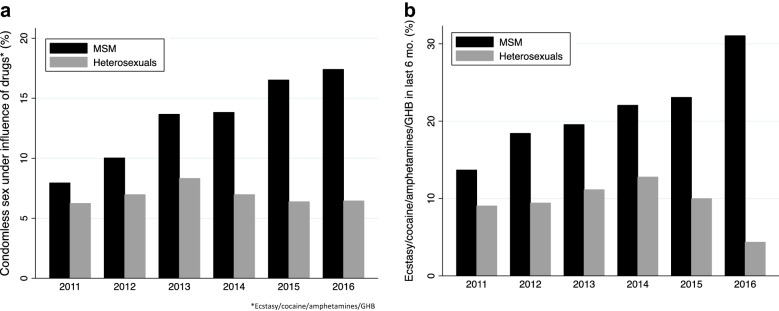
**a** Proportion of Helpcenter attendees reporting ever had sex under influence of ecstasy/cocaine/amphetamines/GHB and **b** use of ecstasy/cocaine/amphetamines/GHB at a party in past 6-months in men who have sex with men (MSM; black) and heterosexuals (grey) between 2011 and 2016

**Fig. 2:**
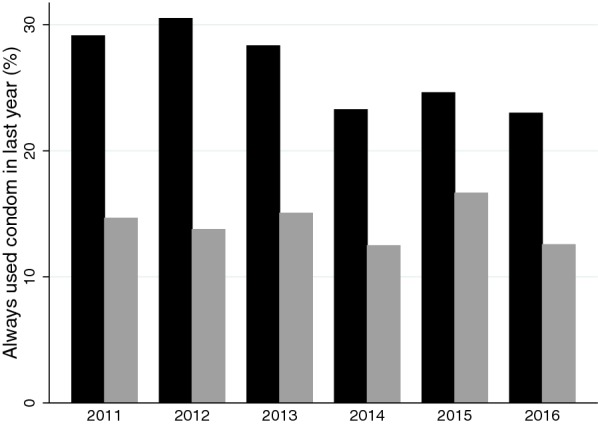
Percent of Helpcenter attendees reporting having always used condoms in the preceding 12 months between 2011 and 2016. MSM are represented in black and heterosexuals in grey

Between 2011 and 2016, there was an increase in the proportion of MSM reporting condomless sex under the influence of all categories of drugs: ecstasy/cocaine/amphetamines/GHB from 29/365 (8.0%) to 114/655 (17.4%), cannabis from 24/365 (6.6%) to 97/655 (14.8%), alcohol from 123/365 (33.7%) to 332/655 (50.7%), and ‘other drugs’ (8/365 (2.2%) to 27/655 (4.1%); all P < 0.05; Fig. 1a). Of the four drug categories that could be taken in the preceding 6 months, there was only a significant increase in use of ecstasy/cocaine/amphetamines/GHB by MSM: 51/373 (13.7%), 77/418 (18.4%), 106/542 (19.6%), 133/603 (22.1%), 96/416 (23.1%) and 9/29 (31.0%) in the years 2011 to 2016, respectively; P < 0.001, Fig. 1b. There was a significant decline in the proportion who reported always using a condom in the previous 12 months (2011: 106/364 [29.1%], 2016: 157/683 [23.0%]; P = 0.002; Fig. 2). Stratifying the drug usage by condom usage in MSM revealed the same increase in condomless chemsex and usage of ecstasy/cocaine/amphetamines/GHB by year in those reporting always, sometimes and never using condoms (data not shown). There were no statistically significant increases in the use of drugs or condomless sex under influence of drugs in the heterosexuals except for an increase in condomless sex under influence of “other” drugs from 10/689 (1.5%) in 2011 to 26/900 (2.9%) in 2016 (P = 0.02).

### Associations with testing positive for HIV and other STIs

A higher proportion of MSM 73/1529 (4.8%) than heterosexuals 29/2992 (1.0%) tested for HIV had a new diagnosis of HIV (P < 0.001). Seventy-seven individuals were diagnosed at their first visit and 25 at repeat visits.

#### Msm

On unadjusted testing in MSM there was no statistically significant difference in age, but HIV-seroconverters were less likely than non-seroconvertors to have a higher education (Odds Ratio [OR] 0.5, 95% confidence interval [CI] 0.3–0.8) and more likely to report 5 or more partners in the previous 12 months (OR 5.5, 95% CI 1.7–18.0), report condomless sex under the influence of ecstasy/cocaine/amphetamines/GHB (OR 6.4, 95% CI 3.5–11.5) and alcohol (OR 2.0, 95% CI 1.1–3.6) and use of ecstasy/cocaine/amphetamines/GHB in the preceding 6 months (OR 6.0, 95% CI 3.2–11.2; Table 1).

**Table 1:** Factors associated with HIV-seroconversion in 1529 men who have sex with men attending the Antwerp Helpcenter (univariate and multivariate associations)

	HIV seroconversion n = 73 n (%)	HIV negative n = 1456 n (%)	Odds ratio (95% CI)	Adjusted Odds ratio (95% CI)
Age—Median, years (IQR)	37 (31–46)	34 (28–43)	1.0 (1.0–1.0)	1.0 (1.0–1.0)
World region			**	*
West Europe	48 (65.8)	1185 (81.4)	Ref	Ref
Sub Saharan Africa	3 (4.1)	47 (3.2)	1.6 (0.5–5.2)	2.0 (0.6–6.7)
Rest	22 (30.1)	224 (15.4)	2.4 (1.4–4.1)	2.2 (1.2–3.8)
Education			*	
Secondary or less	38 (52.1)	529 (36.3)	Ref	Ref
Tertiary	35 (48.0)	927 (63.7)	0.5 (0.3–0.8)	0.6 (0.4–1.0)
Partners in last 12 months (n)			**	
0–1	3 (4.1)	209 (14.4)	Ref	
2–4	17 (23.3)	604 (41.5)	2.0 (0.6–6.8)	NE
≥ 5	38 (52.1)	481(33.0)	5.5 (1.7–18.0)	NE
Missing	15(20.6)	162 (11.1)	6.5 (1.8–22.7)	NE
Condom usage			*	
Always	14 (19.2)	316 (21.7)	Ref	
Sometimes	38 (52.1)	788 (54.1)	1.1 (0.6–2.0)	NE
Never	3 (4.1)	151 (10.4)	0.4 (0.1–1.6)	NE
Missing	18 (24.7)	201 (13.8)	2.0 (1.0–4.2)	NE
Unprotected sex under influence of: ^a^				
Alcohol			**	
No	22 (30.1)	779 (53.5)	Ref	
Yes	27 (37.0)	474 (32.6)	2.0 (1.1–3.6)	NE
Missing	24 (32.9)	203 (13.9)	4.2 (2.3–7.6)	NE
Ecstasy/cocaine/amphetamines/GHB			**	**
No	27 (37.0)	1111 (76.3)	Ref	Ref
Yes	22 (30.1)	142 (9.8)	6.4 (3.5–11.5)	5.7 (3.2–10.4)
Missing	24 (32.9)	203 (13.9)	4.9 (2.8–8.6)	2.9 (1.6–5.1)
Marijuana			**	
No	43 (58.9)	1125 (77.3)	Ref	
Yes	6 (8.2)	128 (8.8)	1.2 (0.5–2.9)	NE
Missing	24 (32.9)	203 (13.9)	3.1 (1.8–5.2)	NE
Use of drugs: ^b^				
Alcohol			**	
No	7 (9.6)	315 (21.6)	Ref	
Yes	31 (42.5)	706 (48.5)	2.0 (0.9–4.5)	NE
Missing	35 (48.0)	435 (29.9)	3.6 (1.6–8.3)	NE
Ecstasy/cocaine/amphetamines/GHB			**	
No	19 (26.0)	885 (60.8)	Ref	
Yes	24 (32.9)	186 (12.8)	6.0 (3.2–11.2)	NE
Missing	30 (41.1)	385 (26.4)	3.6 (2.0–6.5)	NE
Marijuana			*	
No	24 (32.9)	712 (48.9)	Ref	
Yes	10 (13.7)	204 (14.0)	1.5 (0.7–3.1)	NE
Missing	39 (53.4)	540 (37.1)	2.1 (1.3–3.6)	NE

*NE* not entered in multivariable model

P-value: * < 0.05, ** < 0.005; age coded as continuous variable

^a^ Reported having had unprotected sex under the influence of the specified drug

^b^ Reported use of the specified drug in the preceding 6 months

In our multivariable logistic regression model (controlling for age, education and world region of origin) only region of origin (OR 2.2, 95% CI 1.2–3.8) and reporting condomless chemsex (OR 5.7, 95% CI 3.2–10.4) remained positively associated with HIV-seroconversion (Table 1). We conducted three sensitivity analyses. Firstly, repeating the analyses excluding those who first tested HIV positive at non-first visits did not alter the results (data not shown). Secondly, after repeating the multivariate analyses with all those with missing data for the condomless chemsex variable recoded as having engaged in this activity, condomless chemsex remained associated with HIV infection (OR 3.7, 95% CI 2.3–6.2). Thirdly, we reran our multivariable model controlling for age, world region, education, number of partners in past 12 months and condom usage. Condomless chemsex remained associated with HIV-seroconversion (adjusted odds ratio 2.6 [95% confidence interval 1.2–5.7]).

The number of individuals tested for other STIs was lower than for HIV. Unadjusted analysis revealed that condomless chemsex was positively associated with syphilis (OR 3.1, 95% CI 1.6–6.1), gonorrhoea (OR 2.3, 95% CI 1.3–4.0), and chlamydia (2.3, 95% CI 1.3–4.1).

#### Heterosexuals

No positive association was found between reported drug use and HIV (data not shown). There was no statistically significant change in the proportion reporting always using a condom in the previous 12 months (2011: 103/703 [14.7%], 2016: 112/892 [12.6%]; P = 0.580; Fig. 2).

## Discussion

In this single center study, we describe increases in condomless sex associated with various drugs as well as declines in always using condoms in MSM but not in heterosexuals attending the Helpcenter. We also find an association between condomless chemsex and HIV infection in MSM. Our results build on the findings of others which suggest that chemsex may be playing a role in the spread of HIV and other STIs in MSM [2, 5]. One recent bio-behavioural survey of MSM from 13 European cities for example found that 30% reported drug use and 12% the use of two or more drugs during their last sexual encounter. Both use of all drugs and chemsex drugs during last sexual encounter was highest in the only Belgian city in the survey—Brussels. Drug use was associated with both higher risk behaviours and having been diagnosed with HIV [12]. As is the case with our analysis, this study was unable to establish what the direction of the association between drug usage and HIV infection was. These findings are particularly concerning in the context of ongoing high HIV incidence in MSM in Antwerp and Belgium [13, 14].

This analysis is limited by a number of factors. Our sample is a self-selected group of MSM and heterosexuals with higher risk behaviors. As such the results cannot be generalized to other populations. Because risk behaviors tend to cluster, it is possible that the relationship we found between chemsex and HIV is confounded by some unmeasured marker of risk [15]. There was a relatively high proportion of individuals with missing data for some of the questions including drug usage which may have introduced a bias (Table 1). Our questionnaire did not directly assess mephedrone or crystal methamphetamine usage which have been found to be increasingly common drugs used in the context of chemsex [3, 10]. The way drug use was asked did not allow us to disaggregate ecstasy/cocaine/amphetamines/GHB use. Finally it should be emphasized that our findings of an association between sex under the influence of drugs and HIV is limited to the use of the following drugs: ecstasy, cocaine, amphetamines or GHB.

## Conclusions

Whilst our data is neither representative of the general population of Antwerp, nor Antwerp’s MSM population it does reflect the self-reported behavior of a large pool of individuals with elevated risk of acquiring HIV/STI. If our findings of an increase in prevalence of condomless sex under the influence of a range of psychoactive drugs in MSM are reflective of changes in the broader community, then this may be of relevance to our current epidemics of a number of STIs that disproportionately affect MSM [16, 17]. Further studies are required to assess the factors underpinning the rise of chemsex [1, 7, 9]. Concretely, the findings provide further evidence of the importance of asking MSM clients about the use of psychoactive substances during consultations and tailoring interventions such as pre exposure prophylaxis, more frequent STI screening and substance abuse counseling accordingly [3].

## Abbreviations

HIV: Human Immunodeficiency Virus
ITM: Institute of Tropical Medicine
MSM: men who have sex with men
STI: sexually transmitted infection
